# Exploring health care providers’ perceptions about home-based palliative care in terminally ill cancer patients

**DOI:** 10.1186/s12904-019-0452-3

**Published:** 2019-08-06

**Authors:** Heshmatolah Heydari, Suzanne Hojjat-Assari, Mohammad Almasian, Pooneh Pirjani

**Affiliations:** 1 Social Determinants of Health Research Center, Lorestan University of Medical Sciences, Khorramabad, Iran; 2 French Institute of Research and High Education (IFRES-INT), Paris, France; 3 Department of Home-based palliative care, ALA Cancer Prevention and Control Center (MACSA), Tehran, Iran; 4 School of Medicine, Lorestan University of Medical Sciences, Khorramabad, Iran; 5 Department of Community Health Nursing, School of Nursing and Midwifery, Lorestan University of Medical Sciences, Khorramabad, Iran

**Keywords:** Palliative care, Home health care, Advanced cancer, Terminal care

## Abstract

**Background:**

According to the World Health Organization, palliative care is one of the main components of healthcare. As the incidence of cancer is increasing in the world, home-based palliative care can be beneficial for many patients. This study was designed to explore health care providers’ perceptions about home-based palliative care in terminally ill cancer patients.

**Methods:**

This qualitative study was carried out using the conventional content analysis from October 2016 to September 2018 in Iran. Participants were home care providers who were selected using purposive sampling. The data were collected through 18 individual interviews, and a focus group meeting. Data were analyzed based on the method proposed by Lundman and Graneheim.

**Results:**

from the data analysis, 511 initial codes were extracted, which were categorized into the two main categories of challenges and opportunities for home-based palliative care and 10 subcategories. The subcategories of challenges included deficiencies in inter-sectoral and inter-professional cooperation, lack of infrastructures for end-of-life care, challenges related to the management of death, challenges of transferring patients to home, providing non-academic palliative care, lack of political commitment of the government and Spiritual vacuum**.** The category of opportunities included subcategories of cost-effectiveness, moving towards socializing health, and structure of the health system.

**Conclusions:**

Home-based palliative care requires government and health system support. Structural and process modification in the healthcare can provide conditions in which terminally ill cancer patients receive appropriate care in home and experience death with dignity through support of family, friends and healthcare.

## Background

Cancer is becoming more prevalent worldwide. According to the World Health Organization (2018), prevalence of cancer has reached 18.1 million in the world. Recently, cancer-related death rate has been reported about 9.6 million, which makes cancer the second leading cause of death worldwide [1]. These terminally ill patients experience pain and suffering from lower level of quality of life [2].

The World Health Organization considered palliative care as a way of improving quality of life in these patients [3]. Palliative care has a holistic view and takes various dimensions into account in patients including physical, mental, social, spiritual, and economical [4]. In addition, patients have active and dynamic lives until death. This type of care also supports families of patients during illness, death, and even after patients’ deaths, and makes bereavement more peaceful and acceptable for families [3]. Home-based palliative care increases satisfaction and quality of life in patients and their families and also reduces the cost imposed on the health system and re-hospitalization [5–9]. However, palliative care is provided to only 14% of 40 million patients who need palliative care. Around 78% of these patients live in countries with low or moderate income [1].

Iran, as a developing and populous country in the Middle East, faced a high burden of cancer; the prevalence of cancer is progressively increasing making cancer the second leading cause of death. In 2016, 82% of death in Iran was related to chronic diseases, and among these, cancers accounted for 16% of death [10]. Iran is a huge country in which various ethnicities live with language and culture. More than 99% of the population is Shia Muslims and live with minority group such as Zoroastrians, Jewish, and Christians [11].

Healthcare system in Iran is a network system and provides care as primary health care. In the structure of this system, urban and rural health centers is covering specific population, and healthcare is provided at three levels of prevention. Clients can be referred form environmental levels of health centers to general and specialist hospitals [12]. In addition, there are many charity organizations that provide care to chronic and terminally ill patients [11].

Home-based palliative care as a new approach of care don’t have any position in this structure. Private and charity centers provide home-based palliative care [13]. Home care for terminally ill cancer patients should be provided based on the social context and the cultural diversity in the target community [14]. Iran has a unique culture and social context. Implementing any new program in any country requires investigating that phenomenon in order to understand its various dimensions from the viewpoints of the stakeholders. This qualitative study was conducted to explore health care providers’ perceptions about home-based palliative care in terminally ill cancer patients.

## Methods

This qualitative study was conducted using a conventional content analysis approach from October 2016 to September, 2018.

### Study participants

Study participants were selected using purposive sampling method. Participants were knowledgeable and experienced care providers including: hematologists, oncologists, palliative care physicians, nurses, psychologists, religious experts and caregivers.

The inclusion criteria were being involved with home care of patients with advanced cancer at least one year and willingness to participate in the study. The exclusion criteria were being unable and unprepared to participate in the study. Terminally ill cancer patients were those with confirmed diagnosis of cancer, receiving palliative care determined by two oncologists. Participants’ Maximum variation was taken into consideration in terms of age, gender, type of specialty and length of experience in home-based palliative care. As participants were distributed across the country and were not accessible easily, some eligible participants did not announce to take part in the study. Attempts has been made to resolve this limitation through continuous follow up and interview place based on their preferences.

### Data collection

Data were collected through semi-structured individual interviews with 17 people and a focus group meeting consisting of 8 people. Two face to face individual interviews were conducted with one of the participants because of more explaining about questions of study and resolve of some ambiguities in the first interview. In total, 18 individual interviews were conducted. A focus group discussion was held to enrich individual interviews and promote rigor of collected data. The investigator visited a charity institution providing home-based palliative care to patients with advanced cancer in order to have access to the potential participants. First interview was conducted with professionals involved in home-based palliative care. Then, data collection continued in other settings. In order to access authentic and real information, the investigator established a close and direct relationship with the participants. Data collection continued until data saturation achieved, and the participants did not provide new information and no new data obtained from the interviews [15].

A general question was asked, “What is your experience of home-based palliative care?” The researcher directed the interviews towards achieving the objectives of the study through probing questions. During the study, it should be noted that the main question of the study changed in minor ways in light of the obtained information based on the purpose of the study. The duration of each individual interview was between 15 and 40 min, and the focus group discussion lasted about 83 min. All interviews were recorded using an electronic device.

### Data analysis

The data analysis was conducted simultaneously with the interviews based on the method proposed by Lundman and Graneheim [16]. Therefore, each interview was transcribed verbatim immediately. Then the transcriptions were read several times, and initial codes were extracted. Subsequently, related initial codes were merged to form categories based on their similarities. Ultimately, implications and themes of data were extracted.

### Rigor

In order to ensure the accuracy and reliability of the data, the criteria of credibility, transferability, dependability and confirmability were used in accordance with the views of Lincoln and Guba [17]. The researcher established long-term relationship with the participants. After creating the initial codes, the participants’ opinions and comments were used to ensure accuracy and rigor of codes and interpretations. If the codes conflicted with the views of the participants, they were modified. Additionally, the codes were checked by two faculty members who were experts in the field of qualitative research until a consensus was reached on the selected code and categorization. It was also attempted to select participants so that they would have maximum variety in terms of knowledge, experience, and length of service, place of employment, age, and gender.

### Ethical considerations

Objectives and methods of the study were explained to all participants. They were also assured about anonymity and confidentiality of collected data and audio files. Participants had the right to withdraw from the study whenever they wanted. Written informed consents were obtained from the participants. Moreover, the study was approved by the Ethics Committee of Lorestan University of Medical Sciences: the ethics code of LUMS.REC.1394.57.

## Results

In this study, the data were collected through 18 face-to-face interviews (Table 1) and one focus group discussion session. The participants in the focus group consisted of 8 individuals (Table 2).

**Table 1:** Participants’ characteristics

Number	Gender	Rang of Age(year)	Educational attainment level	Work experience (in years)	Position
1	Male	60–65	Specialist physician	30	Active in policy making and home-based palliative care (2 interviews)
2	Male	45–50	Specialist physician	25	Active in policy making and home-based palliative care
3	Female	35–40	B.Sc. in nursing	16	In charge of the coordination of home-based palliative care services
4	Male	50–55	Ph.D. in nursing	26	Lecturer of community health nursing
5	Female	45–50	B.Sc. in nursing	19	In charge of the palliative care unit
6	Male	45–50	Clergy	6	Expert on religious issues of home-based palliative care
7	Female	45–50	General practitioner	18	Palliative care physician
8	Male	40–45	General Practitioner	16	Palliative care physician
9	Female	40–45	B.Sc in social work	11	Social worker in home-based palliative care
10	Female	30–35	B.Sc in nursing	–	Care giver in home-based palliative care
11	Female	20–25	High school diploma	3	Nurse practitioner in home-based palliative care
12	Female	30–35	General practitioner	4	Palliative care physician
13	Male	50–55	Nursing assistant	8	Nursing assistant in the palliative care unit
14	Male	30–35	MSc in psychology	3	Home-based palliative care psychologist
15	Female	25–30	B.Sc in psychology	5	Home-based palliative care psychologist
16	Male	45–50	Ph.D. in nursing	14	Lecturer of community health nursing
17	Female	35–40	Diploma	9	Care giver in home-based palliative care

**Table 2:** Participants of focus group

Number	Gender	Rang of Age(year)	Educational attainment level	Work experience(years)	Position
1	Male	60–65	Specialist physician	32	Active in home-based palliative care
2	Male	50–55	Specialist physician	18	Active in home-based palliative care
3	Female	45–50	Community medicine specialist	19	Representative of the Ministry of Health
4	Male	55–60	Physician	23	Iranian Health Insurance representative
5	Male	50–55	M.Sc. in nursing	30	The manager of a home care institute
6	Male	40–45	B.Sc. in nursing	16	Home-based palliative care nurse
7	Male	50–55	Clergy	26	Expert on religious issues
8	Male	40–45	Ph.D. in nursing	15	Active in home-based palliative care

After analysis, the data were placed in the two main categories including: challenges (7 subcategories) and opportunities of home-based palliative care for terminally ill cancer patients (3 subcategories), which are presented in Table 3.

**Table 3:** The categories and subcategories emerged from data analysis

Main categories	Subcategories
Challenges	Lack of inter-sectoral and inter-professional cooperation
	Lack of appropriate infrastructures for end-of-life care
	Challenges associated with the management of death
	Challenges of transferring patients to their homes
	Providing non-academic palliative care
	Lack of political commitment of the government
	Spiritual vacuum
Opportunities	Cost-effectiveness
	Moving toward socialization of health
	Structure of the health system as an opportunity

### Lack of inter-professional and inter-sectoral collaboration

Inter-professional and inter-sectoral collaboration in providing health services is an inevitable necessity. It is imperative that the healthcare professionals and various institutions have close interdisciplinary and inter-sectoral coordination and cooperation in order to promote health of community. Data analysis indicated that Iranian health system suffers from weaknesses in inter-sectoral and inter-professional collaboration in providing community-based health services, so that different health professions in most cases offer treatment or health promotion programs to patients without coordination with each other. Additionally, various institutions contribute to prevention of diseases and promotion of community health act completely isolated and unlikely to cooperate in implementation of health-related programs. Participants in the study believed that currently, different organizations, such as private home care institutes, the Welfare Organization, and charities provide home care services to patients suffering from advanced cancer. They also mentioned that various organizations such as the media, The Welfare Organization, seminaries, and similar institutions should be involved in and contribute to the provision of health services to terminally ill cancer patients at their homes, so that they can improve the quality of life of these patients and their families. One of the participants mentioned that: “... The Radio and Television as awareness-raising media organization...” [1].

Participants stated that healthcare providers enter individuals’ private space for home care. Healthcare providers and patients are strangers; therefore, safety of both sides could be threatened. It is required that providers entering home consider security issues and coordinate with local security officials. One of the specialist in home care said about providing safety “… the police force in the security sector ... can help us do our work more conveniently…” [1].

One of the healthcare providers about the necessity of providing safety for home care providers stated: “… in home care, we not only work with the patient but also the family … for example, a patient whose family member is mentally ill … Well, the social record of the family should be assessed. Or a family who live in a poor neighborhood … most poor are addicted or traffickers… my mobile was stolen … and hit me …” [17]. Also this care giver stated:“… Nurse who provide home care … they all have fear that it may happen to them such as being assaulted then who is responsible … I also have this fear that if I entered a home, something would happen and no one would be available to help me …” [17].

### Lack of appropriate infrastructure for end-of-life care

In the provision of health services, availability of appropriate infrastructures and models, in accordance with the setting, can be very helpful. Data analysis suggested that the Iranian society has inadequate infrastructure to support terminally cancer ill patients at the community level. Centers such as nursing homes, hospices, and home care for supporting these patients until the time of death play an insignificant role in managing this process.

Regarding the necessity of hospice centers in Iran, a palliative care specialist said:“ …The best condition for these patients is hospices that should be existed … when the family is not able to care the patient at home, there should be places that an individual can be referred and spend last time without healthcare …” [1]. Another participant stated about problems of telemedicine in Iran“… telemedicine can be helpful in home care but the internet is not available anywhere in Iran… if there was a strong net, many works could be performed… but unfortunately, there is poor internet…” [2]. Moreover, there are no laws, rules, and guidelines that act as a roadmap for healthcare staff and patients’ families. Data analysis indicated that home-based palliative care is a nascent service in Iran, and there is not a good model for providing these services. Furthermore, existing guidelines are not comprehensive and complete. In many cases, these guidelines have been designed for a specific area of the country or a given university. “There are no proper guidelines drawn based on scientific principles approved by academic communities. One of participants stated:”... In Iran ... a grade 3 or 4 cancer patient still undergoes chemotherapy until the last moment... We do not have a comprehensive and homogeneous model for providing services to these patients. ...“ [2]. Also One of the psychologist in palliative care expressed lack of infrastructure in this field ”… unfortunately, position of psychologist is not clear…, “position of psychologist is not identified … we have problems in terms of hardware. In terms of software, it should be necessary to write protocols and guidelines for psychological services …” [14]. A palliative care specialist stated the importance of providing infrastructure“… heath care system, the Ministry and universities have not yet reached to this poor point and missing link …” [1].

### Challenges associated with the management of death

Death is an inevitable phenomenon, which should be considered an important part of the process of life. Data analysis suggested that people are horrified by death and self-censorship is common among them. They suppress death into the unconscious, and they postpone dealing with this process as long as they can. Religious expert stated about fear of death in Iranian society “…we have censored death in the society …… in schools…, at universities…, nothing is said about death…Scholars and students do not know about death…have you ever seen a program on TV... These make … people consider death as monster as they are not familiar with death …This censor of death leads to have problems in discussion of death with patients…” [3]. another participant stated that “…in Iran, the biggest problem is the issue of death…there is no guideline for management of death at home …,we do not have laws for terminally ill patients ...We do not have laws about how the patients should die, where they should die ... about which patients should be resuscitated and which ones shouldn’t ...unfortunately, they still do not understand the concept of dying ... It is very difficult to make a patient’s relatives accept and understand the patient’s death ... “ [1]. And religious expert said:”… some patients have not good memory about relative’s death … you need to remove this memory from his/her mind and ensure that death means transfer …” [3].

### The challenges of transferring patients to their homes

The patient’s place of residence is one of the places where the patient can receive high quality services. Data analysis indicated that the health system and clients have to face numerous problems with the provision of home care services. Also data analysis indicated that Iran is a country with specific beliefs based on which, in many cases, service recipients insist that the patient should stay at hospital until she/he dies and receive specialty pharmaceuticals and, even in some cases, the hope that the patient survives for a few more days encourages the family to bear substantial health care costs. In some cases, the patients’ families insist that the patients die on hospital beds. Furthermore, data analysis indicated that Iranians prefer to receive services from specialists in most cases, and are not as willing to receive health care services from general practitioners or nurses at home. A specialist physician said: “... Unfortunately, Iranians are still not used to accept nurses and general practitioners and home care doctors ...” [1]. Another participant stated: “... Differences in opinion overshadow management of patient’s condition until the last moment ...to overcome this inconsistency, an appropriate culture should be created...” [2].

Some health professionals believe that transfer of patients to home means confirmation of their death, and if the patient is transferred to home, he/she will be deprived of many health services. Participants mentioned, most patients with incurable disease die at hospital, and to transfer the patient to their home, first it is necessary to conduct family meeting sessions and because many family members do not agree with transferring the patient, obtaining the family’s consent is the most difficult part of the transfer to home. One of the participants about problems of transferring patient to home said: “… in Iran, no one makes decision for transferring patient to home except the patients’ relatives … here, the biggest issue is patient’s relatives … even if the cost was free, 7 out of 10 patients do not accept to be cared at home… if they discharge, they will refer to other hospitals …” [1]. Also religious expert said: “... some patients resist to be transferred to home due to either conditions or death …” [6].

The manager of one of the palliative medicine institution said: “… during the transfer of a patient to home, her/his relative have bizarre expectations, they think we can save them … while they know patient’ condition…” [3]. And one of the palliative physician said: “...even in come, some relatives do not want to accept that the patient may pass away… he/she may heal, may improve…” [12]. This participant also said about an unpleasant experience of a family who insisted on therapeutic interventions till the end “… a patient was in coma, we intubate as her son insisted then he insisted to extubate… I said we have no right and resuscitation must be performed … we cannot do that …” [12].

### Providing non-academic palliative care

Incompetence of professional health care providers offering home care to terminally cancer patients was another issue that the participants pointed out. They said that providing home care services to terminally ill cancer patients is not taught in courses and curriculums in health education, and graduates of these fields, especially physicians and nurses, are not familiar with different dimensions of this care approach. Religious experts and psychologists do not know anything about the concept of palliative care, and do not have skills to deal with these patients and calm them down. One participant stated: “..... End-of-life care of advanced cancer patients should be systematized and taught at university level ....” [2]. another participant said: “... All health personnel, even clerics, nurses, and psychologists, should take courses from three to six months... for their attitudes to change ...” [1].

The participants pointed out nurses are the most important health care professionals, who should be active in providing care and management services to the patient and their family from the moment the patient is admitted to the last moment. As training of nursing staff is hospital-based, nurses should fully understand and be familiar with the mission, goals and skills needed to provide home care health services to terminally ill cancer patients. One of the participants stated, “…nurses, as the most important members of health team, are not recognized outside hospitals, and are unfamiliar with providing health services at the community level…compensations and benefits for community-based health services are far less significant than those of providing services at treatment centers... therefore, nurses’ needs must be met both financially, spiritually and educationally, and in terms of level of education and social status ...” [2].

### Lack of political commitment of the government

The government’s support and cooperation of authorities is necessary for success of programs in the health system. Data analysis suggested that the health system has been addressing this issue in recent years. However, some management layers of the health system are still not familiar with or disagree with this approach to the delivery of health services. Data analysis showed that in order to provide home-based health services to terminal cancer patients, the health system should have a community-oriented perspective and provide conditions that make the patients unwilling to be hospitalized and occupy hospital beds. One of participants stated that, “….. Under the current conditions, the health system assigns hospitals pivotal importance, and various health professionals have a treatment-oriented perspective….” [1].

In addition, data analysis suggested that one of the challenges of providing home care services to terminally ill cancer patients was lack of insurance coverage of this type of care that causes many problems for patients, their families, and service providers.

Analysis of data showed home-based palliative care has no place in health care system in Iran, in this regard, one of the participants said: “…This care should be include in the structure and allocate a specific fund … where in the structure, who are involved, under what conditions and standards…” [4].

### Spiritual vacuum

Data analysis showed that spiritual aspect as one of the dimensions of palliative care that has been neglected in the end-of-life care of cancer patients. Study participants expressed that although Iran is a Muslim country in which spirituality is dominant, the potential of religious experts is not fully utilized to improve quality of life in advanced cancer patients. They pointed out that religious experts could offer counselling based on patients beliefs, accompany them from hospital to home, and help patients and their families be calm and peaceful. They also noted that religious experts are capable of setting the stage for a peaceful death for patients, and offer companionship to families in the face of grief and afterwards.

One of the psychologist said: “… spiritual care and psychological consultation are the missing links in healthcare …” [14]. Another religious expert, about necessity of spiritual consolation stated, “... We must support the family after the death of the patient, because their peace of mind is disrupted ... We should manage the patient’s death and support the family and relatives….” [3].

### Opportunities

#### Cost-effectiveness

Data analysis showed that home care for terminally ill cancer patients can be cost-effective for both families and the health system. Patients can also receive high quality services, which leads to improvement in patients and families’ quality of life.

Participants stated that home care for patients with cancer could reduce healthcare cost through unoccupied hospital beds. If terminally ill patients pass away at home, bed occupation in critical care units is reduced, and these beds could be provided to patients who have a higher life expectancy. A religious expert said: “… suppose that two patients are admitted to the emergency department, one of them is car accident and the other is terminally ill cancer patient… it is rational to whom the bed should be provided …” (Clergy in focus group). Participants mentioned that maintenance of hospital beds cost hundred dollars. Avoiding of hospitalizing patients with cancer in critical care units decreases establishing further beds at hospital. A participant from nursing society said: “… a patient who can be cared at home occupy ICU bed at hospital…ICU bed cost so much … home care can decrease the cost so much…It is beneficial for people, healthcare and patients …” [4]. Also, one of the oncologist in home care field said:…critical care beds should be used for saving patients … rather than patients with cancer die on it … if patient with cancer die at home, at least 10% of hospital beds are released… establishing critical care bed costs so much … transferring patients to home results in decreasing the cost of establishing and maintenance of critical care beds…a patient who is cared at home experience fewer complications which is beneficial for both insurance and patients... [1].

Participants mentioned that this care approach is cost effective for families so that they need to visit patients during hospitalization. In may continue for all family members for days, weeks and months. One of the home care physician said:“… home care reduces family members’ trips and traffic jam...” Family members do not need to leave work to visit“… [3]. They also mentioned that given busy hospitals and no visit in ICUs, family members’ dignity is maintained so that one of the participant said:”… the quality of services is better at home … dignity of caregivers and family is maintained better… [16].

#### Moving toward socialization of health

Data analysis has indicated that in recent years the health system has demonstrated a firm resolve to move towards socialization of health, so that progress has been made towards using nongovernmental potentials, like philanthropists contributing to health projects. Study participants noted that primary, secondary and tertiary preventive services can be provided to terminal cancer patients using the nongovernmental potentials of charities. They stated that in the Iranian society, presence of philanthropists and benefactors is a potential that the health system could use to improve quality of life in patients with cancer. Health care can set up centers for these patients in order to provide high quality services to patients at the community level. Many non-governmental organizations have recently invested considerable amount of human and financial resources in healthcare, and most of such organizations provide home care services to terminal cancer patients. Study participants also pointed out that establishment of home care nursing centers in the health system in recent years has been a move towards community-oriented health services. About the delivery of home care services to terminal cancer patients, an employee of a charity organization said, “... We have been providing free services to these patients for about 5 years ... In addition to improvements made to the quality of life of individuals and families, a large number of patient died at home. ...” [1].

#### Structure of the health system as an opportunity

Data analysis indicated that Iran’s health system has the potential to provide community-based services to terminal cancer patients. Study participants noted that health services are readily provided to everyone based on the principles of primary health care in urban and rural areas. There is also a referral and a family physician system that has been created at the rural level, a can be defined as part of the family physician program for providing care for terminal cancer patients. A specialist active in the field of home health care services about considering structure of the health system in Iran as a potential mentioned, “... In health centers, where the population under coverage is known ... even in each village, the rural health center can offer interventions ... the structure of this system can be scrutinized...” [1]

## Discussion

In this study, perceptions of health care providers were explored regarding home-based palliative care. Data analysis showed that providing healthcare poses many challenges to patients in the end-stage of cancer. However, there are also potentials, which can be developed for home-based palliative care.

One of the problems of home-based palliative care was lack of inter-professional and inter-sectoral collaboration. Seow et al. pointed out that inter-professional cooperation is an element that could enhance quality of home-based palliative care. Thus, there should be coordination and cooperation among different elements of health care services including home, hospital, primary health care physicians, and health centers [18]. When health care providers work closely together as team members, they can share patient information; consequently, they avoid unnecessary parallel work and offer comprehensive services.

A variety of organizations should cooperate for providing community-based healthcare. Given the primary healthcare in Iran, different organizations should cooperate to maintain and promote heath. In community-based care, professionals enter homes and community; therefore, safety should be taken into consideration. In addition to security services, healthcare providers can use capabilities of local police.

Infrastructure for end-of-life care is one of the issues that needs to be resolved for providing home-based palliative care services in the community. LaVigne et al. pointed out infrastructure is required including access to analgesic drugs, inclusion of palliative care in the educational curriculum of health personnel, and access to financial resources [19]. In addition to training qualified personnel and allocating resources for home-based palliative care services, health systems should investigate appropriate models and guidelines in accordance with the context and setting in which the services are provided. The framework and roadmap can be used for providing high quality services, which require the political commitment of the government for establishing the infrastructures [20]. In addition to providing software, home-based palliative care requires preparing appropriate physical environment in patients’ homes. Medical equipment and medications should also be provided to patients and families. Thus, they can spend last days without concern about cost of equipment with their families.

Results showed that at the community level, the death anxiety is common in patients with advanced cancer, and try to postpone the time of death as long as they can. However, the WHO emphasizes that death should neither be speeded up nor postponed for terminally ill patients, so that they go through the natural process of life and death [4]. Sheng et al. mentioned that health staff should have accurate information on the remaining time for the patient, prognosis, symptoms the patient will encounter, outcome and complications of resuscitation, and patient’s wishes, so that they can provide comprehensive information to the family and be able to overcome the barriers to the patient’s natural death [21].

Studies have shown that social support [22] and religious believes [23] could affect death anxiety. Religious people have more positive attitude compared to nonreligious people. Given cultural and religious context in Iran, it seems that religious people could adapt to disease-related pain and sufferings. Social networks and close relationships between people could increase self-confident and neutralize adverse events. While disturbance in such relationships could increase concerns in patients. On the other hand, religious experts and psychologists have an important role in coping with problems and tension resulting from disease. As individual may experience psychological disorders including loneliness, anxiety, and loss of life meaning; therefore, death anxiety increases. Spiritual and psychological consultation in the early days of illness and reinforcing family relationships could prepare an individual for pace death.

Data analysis indicated that moving towards socialization of health and existing a primary health care program in the health system could be considered as a potential for promoting home-based palliative care for end-stage cancer patients. Additionally, the findings suggested that using nongovernmental potentials, such as charity and volunteer organizations, could be helpful in improving home-based palliative care programs. In this regard, the WHO has emphasized that health systems should offer palliative care [3]. However, there is a wide gap between this priority of the WHO and people access to palliative care in the low- and middle-income countries. The majority of people in these countries have no access to palliative care [4]. One of the most important barriers to access this kind of care is limited financial resources in health systems. Some studies have shown that the cost of this kind of service accounts for 10% of the total expenditures of health system in the Netherlands [24], 13% in the United States [25], and 26% in Great Britain [26]. In line with the findings of this study, an international study showed that, palliative care is funded by charitable, public, and private organizations in most developed countries [27]. Similarly, the Can Support model, which is currently used for providing home-based palliative care in India, is sponsored by the NGOs and has reached considerable achievements in delivering high quality and cost-effective health services to terminally ill patients [28]. Therefore, it seems that supporting NGOs by the health system as the organization in charge of health care issues in the country, can deliver higher quality services to terminal cancer patients.

Results suggested that existing community-based services in the structure of the health system is an element that can improve home-based palliative care programs. In line with the findings of this study, Kim et al. proposed a high quality services for end-stage cancer patients using a community based palliative care service model [29]. In this regard, Iran’s health system follows a network model, which is provided as primary health care (PHC). The most important principles are centrality of equity and easy access to health services. The structure of this system is so that urban and rural health centers are in charge of health of a neighborhood; therefore, home-based palliative care can be placed in this structure. Reciprocal access is provided for healthcare providers and terminally cancer patients at home easily.

## Limitations

Among the limitations of the study, we can point out the dispersion of home-based palliative care practitioners throughout the country, which made it difficult to access and interview them in person. However, with the persistence and perseverance of the authors, and setting accessible locations for the interviews, some of the existing barriers were overcome.

## Conclusion

This study revealed that there are many challenges in providing care to terminally ill cancer patients. Home-based palliative care can be improved through addressing these challenges. The results of this study can be helpful in clinical, education and policy making fields of home-based palliative care. In the clinical field, healthcare providers could cooperate interdisciplinary and extra disciplinary with other healthcare providers and organizations. Death anxiety can be reduced through spiritual experts and psychologists. In education, palliative medicine courses should be included in the curriculum of nursing, medicine and rehabilitation, which help students be familiar with dimensions of palliative care. They also can have competence of providing healthcare to terminally ill cancer patients at home. In policy making, the government could support this healthcare program and define these services at the structure of primary healthcare so that patients have access to palliative care at home.

## Data Availability

This study conducted by qualitative method, audience and text file are available from the corresponding author if needed.
